# Peplau's theory for telenursing with the family of patients with COVID-19

**DOI:** 10.15649/cuidarte.3139

**Published:** 2024-05-22

**Authors:** Andressa Oliveira das Chagas Morais, Ana Lívia Castelo Branco de Oliveira

**Affiliations:** 1 Centro Universitário Santo Agostinho, Teresina-PI, Brazil. andressaoliveiramorais@hotmail.com Faculdade Santo Agostinho Centro Universitário Santo Agostinho Teresina-PI Brazil andressaoliveiramorais@hotmail.com; 2 Centro Universitário Santo Agostinho, Teresina-PI, Brazil. analiviacbranco@hotmail.com Faculdade Santo Agostinho Centro Universitário Santo Agostinho Teresina-PI Brazil analiviacbranco@hotmail.com

**Keywords:** Interpersonal Relationships, Telenursing, Nursing Care, COVID19, Relaciones Interpersonales, Teleenfermería, Cuidado de Enfermería, COVID-19, Relações Interpessoais, Telenfermagem, Cuidados de Enfermagem, COVID-19

## Abstract

**Introduction:**

The care challenges brought about by the COVID-19 pandemic required nurses to have new skills in coping with health demands. In addition, this professional emerges as an important tool through the therapeutic relationship. Thus, they can be seen as a bridge of support between family members and patients.

**Objective:**

To know the perspective of nurses on telenursing as therapeutic in the context of family members of patients with COVID-19 and to discuss in the light of Peplau's theory of interpersonal relationships.

**Materials and Methods:**

Cross-sectional, exploratory study with a qualitative approach to the data, carried out in a private general hospital in Teresina-PI, in 2022, with a semi-structured interview. Followed by a reflective structure with the use and philosophical theoretical construct of Hildegard Peplau's theory of interpersonal relationships, from 1952.

**Results:**

Nurses perceive remote assistance as an important tool for meeting the psychological and social needs of the family of patients with COVID-19. On the occasion, the nurses look at the patients' relatives experiencing the therapeutic relationship through information mediated by telenursing, which included the description of the main daily care, among other information.

**Discussion:**

Peplau's theory of interpersonal relationships gives meaning to the experience of telenursing from the perspective of nurses.

**Conclusion:**

The perspective of nurses on the remote therapeutic relationship with family members of patients diagnosed with COVID-19 infection involved bonding and welcoming as points of development of trust, which brought positive results to family members, given the uncertainties experienced during the pandemic.

## Resumen

**Introducción:**

Los desafíos de salud planteados por la pandemia de COVID-19 exigieron nuevas habilidades de las enfermeras para enfrentar las demandas de salud. Además, este profesional emerge como una herramienta importante a través de la relación terapéutica. Por tanto, pueden verse como un puente de apoyo entre familiares y pacientes.

**Objetivo:**

Comprender la perspectiva de los enfermeros sobre la teleenfermería como terapia en el contexto de familiares de pacientes con COVID-19 y discutir a la luz de la teoría de las relaciones interpersonales de Peplau.

**Materiales y Métodos:**

Estudio exploratorio transversal, con enfoque de datos cualitativos, realizado en un hospital general privado deTeresina-PI,en2022, con entrevistas semiestructuradas. Seguido de una estructura reflexiva utilizando el constructo teórico filosófico de la teoría de las relaciones interpersonales de Hildegard Peplau, de 1952.

**Resultados:**

Los enfermeros perciben la asistencia remota como una herramienta importante para satisfacer las necesidades psicológicas y sociales de las familias de los pacientes con COVID-19. En esta ocasión, los enfermeros observan a los familiares de los pacientes que viven la relación terapéutica a través de informaciones mediadas por la teleenfermería, que incluye una descripción de los principales cuidados diarios, entre otras informaciones.

**Discusión:**

La teoría de las relaciones interpersonales de Peplau da sentido a la experiencia de teleenfermería desde la perspectiva de las enfermeras.

**Conclusión:**

La perspectiva de los enfermeros sobre la relación terapéutica a distancia con los familiares de los pacientes diagnosticados con infección por COVID-19 involucró el vínculo y la acogida como puntos de desarrollo de la confianza, lo que trajo resultados positivos para los familiares, dadas las incertidumbres vividas durante la pandemia.

## Resumo

**Introdução:**

Os desafios assistenciais trazidos pela pandemia por COVID-19 exigiram dos enfermeiros novas habilidades no enfrentamento de demandas de saúde. Além disto, este profissional surge como importante ferramenta através do relacionamento terapêutico. Assim, podem ser vistos como uma ponte de apoio entre familiares e pacientes.

**Objetivo:**

Conhecer a perspectiva de enfermeiros sobre a telenfermagem como terapêutico no contexto de familiares de pacientes com COVID-19 e discutir à luz da teoria das relações interpessoais de Peplau.

**Materiais e Métodos:**

Estudo transversal, exploratório, de abordagem qualitativa dos dados, realizado em um hospital geral privado em Teresina-PI, em 2022, com entrevista semiestruturada. Seguido de estrutura reflexiva com utilização e construto teórico filosófico da teoria das relações interpessoais de Hildegard Peplau, de 1952.

**Resultados:**

Os enfermeiros percebem a assistência remota como importante ferramenta para o suprimento de necessidades psíquicas e sociais da família dos pacientes com COVID-19. Na oportunidade, os enfermeiros lançam o olhar sobre os familiares dos pacientes vivenciarem o relacionamento terapêutico por meio das informações mediadas pela telenfermagem que incluía a descrição dos principais cuidados diários dentre outras informações.

**Discussão:**

A teoria das relações interpessoais de Peplau dá sentido à experiencia da telenfermagem na perspectiva das enfermeiras.

**Conclusão:**

A perspectiva de enfermeiros sobre o relacionamento terapêutico remoto com familiares de pacientes diagnosticados com infecção por COVID-19 envolveu o vínculo e acolhimento como pontos de desenvolvimento de confiança, o que trouxe resultados positivos aos familiares, diante das incertezas vivenciadas durante a pandemia.

## Introduction

The care challenges brought by the COVID-19 pandemic has required new skills from nurses to face more complex health demands, such as those of patients in critical care. Patients and family members, in turn, when dealing with the uncertainties brought by the virus, found themselves permeated by the fear of losing their family members^1^. Therefore, nursing emerges as an important tool through the therapeutic relationship. Thus, they can be seen as a support bridge between family members and patients.

In this scenario, Hildegard Peplau makes contributions highlighting the therapeutic relationship that prioritizes the shared relationship between the nurse, patient and community, which points to the concept of holistic care. Therefore, the needs met, such as dialogue, the patient's need to leave their bed and walk through the hospital corridors, or talk to the family, are attitudes that promote personal growth and the care process of this relationship^2^.

With this, theorist Peplau confirms that the relationship between nurse-patient-community generates mutual growth with all the experiences lived by them, that is, it is nursing responsible for the process of care and well-being^3^.

Thus, the nurse behavior who provided care to the patient with COVID-19 was no different, as due to the uncertainty about the hospitalized patient's situation, the family's levels of stress and anxiety increased. This way, scholars provide evidence about the work of nurses who maintained communication with the family to talk about their loved one's situation and how they were recovering, thus making the family appear calmer and more hopeful^4^.

This way, nursing in the exercise of interpersonal relationships says a lot about the attitude exercised with patients and their families, and thus, when clarifying doubts, offering advice, transmitting knowledge, participating in the treatment and recovery process. Such attitudes help the patient to face and recognize the illness process, encouraging them not to give up on treatment and not to alleviate sadness and anxiety^5^.

It is noted that the change in care perspective from the hospital-centered medical model to the health determinants model allows for a broader view of the patient's needs, and in the case of the exercise of the therapeutic relationship. Therefore, there is a special focus on mental health needs that contribute so much to coping with physical illnesses such as COVID-19, as it is a disease that is still being revealed by the scientific community. Thus, the topic emerges with relevance and nursing gains dialogue with other sciences that study the nature of human and social relationships.

Therefore, the objective of this study was to understand the perspective of nurses from a private general hospital in Teresina-PI on telenursing as a therapy in the context of family members of patients with COVID-19 and to discuss it in light of Peplau's theory of interpersonal relationships.

## Materials and Methods

This is a cross-sectional, exploratory field research, with a qualitative approach and based on data^6^. The data was collected using a voice recorder application on the researchers' smartphone. They were submitted to manual transcription in the Microsoft Word 2007 program and stored in a folder for later analysis of the statements. Followed by a reflective structure using a philosophical theoretical construct in the theoretical light of Peplau in the theory of interpersonal relationships. The data was organized and stored in Mendeley Data^7^.The Consolidated criteria for reporting qualitative research (COREQ) checklist for qualitative methods was used as a basis^8^.

Hildegard Peplau's theory of interpersonal relationships describes the professional nurse with the potential to identify difficulties and problems with their client/patient and family, and also to resolve them in this process, the link^3^^,^^9^.

With this, it is concluded that Peplau's theory brought great contributions to nursing since the professional's attention is focused on the patient in a different and unique way aimed at good quality mental health^10^. Therefore, it was chosen as a matrix for interpreting the qualitative data evidenced in interviews with nurses who experienced telenursing as a therapeutic resource with family members of patients with COVID-19.

Data collection was carried out between May and August 2022 in a general reference hospital for adults providing COVID-19 care in the city of Teresina, Piauí, Brazil. The service has 5 Intensive Care Units (ICU) for adults, which, during the pandemic, provided remote medical and nursing information services to patients' families. Furthermore, ICUs were used for patients with complications from COVID-19; Visits were only permitted after the second wave of the disease and if the patient was conscious and oriented. As a result, assistance was provided to an average of 10 families per ICU coordinator, as each nurse was responsible for an ICU for better care.

Participants were selected according to an intentional sample. The inclusion criteria were nurses working for at least 6 months in Intensive Care Units, who worked in the care of critical patients, as well as local managers and supervisors. The exclusion criteria were nurses removed or redirected from the target sector due to contamination or comorbidities; nurses who refused to sign the Informed Consent Form (TCLE); nurses who did not give feedback to the researchers about the interview meetings.

Five (5) interviews were carried out, which varied between 8 and 18 minutes, with the date and time agreed via WhatsApp between the interviewee and researcher. The audio recordings were made by two researchers using a smartphone device. Each participant chose their interview location in a reserved space, in the institution itself and free from the presence of third parties. The afternoon shift was adopted by most of the interviewees due to the reduced flow of activity they carried out, being guided about the availability of time and freedom of speech as needed.

Data collection was mediated by two semi-structured interview scripts constructed from the theoretical framework adopted with pre-testing of the sound quality in the recording. The script adopted sociodemographic and occupational characterization variables. Then the question script: How did the remote assistance happen? How was the feedback from patients' families given to the remote nursing assistance?

After data collection, the statements were manually coded into E1, E2, E3, E4 and E5, in a table created in the Microsoft Word program, allowing the anonymity of the participants and the context was discussed together with the scientific literature on the topic. Therefore, the statements were organized by similarities, then content analysis was carried out to correctly extract the relevant concepts and evidence that resembled the perspective of Pelplau's theory of interpersonal relations^3^^,^^11^.

The study was approved by the Research Ethics Committee of the Federal University of Piauí (UFPI) through Opinion No. 4,987,111. The planning and execution were supported by National Health Council Resolutions No. 466/12, 510/16 and 580/18, when the criteria for research carried out with human beings were met^12^^,^^14^.

## Results

There were 5 professional nurses, all female and with experience in care and technical responsibility (management) of intensive care units in the aforementioned service. The interviews provided evidence about the perspective of nurses who implemented telenursing during COVID-19, with the statements demonstrating the importance of the therapeutic relationship with the family of patients affected by this disease.

Telenursing emerged from the moment the unit's nurses observed the impossibility of families making daily visits and the high risk of contagion from the COVID-19 virus. As a result, the families showed a lot of anxiety, which was mitigated by the information provided by the nurses when they got in touch via phone calls.

According to the nurses, during the call they made to bring information to family members, especially about basic needs and assistance actions provided, families demonstrated feedback and felt reassured in their distress, in addition to nurturing confidence in the specialized care provided to their family member:

“The family members liked it a lot, because they were very anxious about not knowing certain news that the doctors didn't give [...]”: E2.

“[...] we had good feedback from families, I think if we could talk to most of them today, we would have favorable feedback even from those who died, they really felt more prepared [...]”: E1.

“They really enjoyed our connection [...], they asked for test results, they always asked if the patient had opened their eyes, if they had “peeed”. They really asked about physiological needs, whether I was taking a shower and they were very receptive”: E4.

“[...] they offered snacks to the teams due to our contact with them. Imagine that throughout the hospitalization we were the bridge that connected the patient to the family”: E4.

The positive evaluation seems to be related to the nurse's interest in forwarding the patient's information to the family, to include the family in this relationship and not just generate attention focused on the patient. Given the bond between professionals and families, there were scores on aspects of nursing aimed at the humanization of care and holistic care, as evidenced in the statements:

“Nursing already has that image of being humanized. That whole empathy thing. So they knew that we were going to give more details about the patient and they were already worried when we were a little late in making the call [...]”: E2.

“So, with the aim of strengthening these ties with the family, we made daily calls, passing on a summary of the patient's clinical condition to their guardian or to another family member designated by the guardian or by the patient themselves”: E5.

The bond with family members is related to the sense of gratitude on the part of family members, which marked the nurses' perception. When patients were discharged, they always thanked and wanted to get to know the professional who provided the assistance better:

“There was a family member who, even after the patient was discharged, would contact us and reciprocate by sending snacks to the nursing team”: E2.

“[...] we ended up getting closer to the family and they understood that we were providing care in a humane way, so it arose from these families’ needs to understand that we were providing care in a more humane way”: E3.

This form of reception for the family generated greater peace of mind even when the patient died, as they received full care from the medical and nursing team. They felt welcomed, because even though they were far away, the nurses kept them close, as seen in the participants' speech:

“[...] some people still had that bond after discharge, even those whose patients had an unfavorable death outcome, so they felt safe and didn’t have that stigma from the hospital”: E2.

“There are people who, at the time of COVID-19, when we had remote assistance, the family where the patient had contact with me and even the family where the patient died are very grateful; They still talk to me, send me messages, in short, we really created this bond of friendship and trust”: E3.

In addition to the bond created, the professionals interviewed reported the experience as unique and positive, with the statements highlighting the importance of the nurse's relationship with the patient's family, which characterizes positive bonding experiences for the family member and the professional who provided remote assistance.

## Discussion

The participants' statements brought data that highlighted telenursing in institutions and the therapeutic possibilities constructed by nursing by strengthening the therapeutic bond. Therefore, the relationship between family members and nursing is emphasized, in order to demonstrate gratitude for the work of the teams that cared for patients.

The statements point to therapeutic communication exercised through telenursing. The importance of communication, a global goal for patient safety, has been the focus of studies with professionals in Intensive Care Units who cared for patients diagnosed with COVID-19. The humanized treatment provided by nurses in the COVID-19 Intensive Care Unit must prioritize comprehensiveness, be holistic in nature, involving the patient and family, subsidized by communication with the aim of promoting improvements to the patient^15^^,^^16^.

In the statements collected, there is an emphasis on the positive balance of strengthening the bond between professionals and family members in order to plan actions together during the care process. This characteristic of care was also noticed and reflected in another study that concluded that active family involvement shows improvement in the patient's general condition^17^.

Thus, aspects related to humanization were part of the perception of the participating nurses who reported the experience of telenursing as unique and remarkable and were precursors of the therapeutic bond.

Then, it is important to highlight the support coming from the team that takes care of your loved one, with actions adapted to each person's reality, providing humanized support, welcoming care, generating interpersonal relationships and a lot of dialogue^18^.

Teen nursing in the context of the nurse-family relationship was also the focus of study in a health unit in the city of Manaus, Brazil. The link helped control crowds of people outside the unit waiting for news. This contact via phone call was very well arranged in terms of days and times^19^.

Among the actions to carry out this contact are: analyzing personal data, checking medical records, making a call to the responsible person registered in the patient's medical record, making sure that the person who will receive the news is in the appropriate place and time, listening to them carefully and request professional emotional support^20^.

This study also highlights that there was a sense of gratitude even in negative outcomes for the patient, such as in the case of deaths. For assistance to take place within the patient's entirety and in accordance with humanization, it is necessary for the Intensive Care Unit nurse to carry out good planning, and this includes the family in the execution of care, aiming for a good recovery of the patient. This attitude emanates from family members and patients a feeling of gratitude^19^.

The study of interpersonal relationships in care contexts has been explored in the light of theoretical constructs^21^. In this sense, it is suggested to think about the therapeutic relationship provided by nursing from the perspective of Pepalu's theory of interpersonal relationships.

Hildegard Peplau's theory of interpersonal relationships describes the professional nurse with the potential to identify difficulties and problems with their client/patient and family, and also to resolve them, in this process the bond^9^. Peplau's theory brought great contributions to nursing since the professional has his attention focused on the patient in a different and unique way aimed at good quality mental health^10^.

According to the findings in this study, the phases that make up the aforementioned theory can be seen in a transversal way. The identification phase could be perceived as the family identified with a nurse, placing trust and developing a therapeutic bond^22^. In helping them to obtain information about the health situation of their loved one, and through the telephone call, this exchange of affection where the nurse answered all doubts, strengthened positive thoughts, helped the family in facing the problem and, in addition, conveyed tranquility.

The first phase of the theory of interpersonal relationships is the orientation phase, it happens when the patient seeks help from the nurse to solve a problem or need and this approach emerges with the family^3^^,^^9^. Therefore, the phase is perceived at the moment of social isolation, The use of technology in nursing was what brought this professional's family closer together. The desire was to alleviate the fear and distress of distancing. In this aspect, the nursing bulletin represented humanization.

In the identification phase, the patient calls on the nurse he trusts to help him with any difficulty and the professional motivates and encourages him to deal with the therapeutic path^3^^,^^9^. This phase was expressed by the family members' sense of gratitude for the nurses' welcome. The information was about care related to the human needs of the loved one, such as eating, breathing, mobility, skin integrity and others.

In phase three, exploration, the nurse helps the patient solve their problem using dialogue and tips to clarify and teach what the patient can do about their dependence or independence^3^^,^^9^. Here, the importance of communication between professionals and family members is noted, which may involve educational processes, in addition to nursing interventions such as assistance in coping with anxiety.

And in phase four, resolution, the bond between nurse and patient is broken after the patient solves their problem and/or achieves independence^3^^,^^9^. At this moment, the patient's outcome was recovery or death, which still maintained the sense of gratitude to the professional, as telenursing made it possible to monitor the therapeutic trajectory.

Given these reflections, Peplau's theory of interpersonal relationships brings meaning to the experience of telenursing from the perspective of nurses, demonstrating the potential of nursing to promote the health of patients and their families, which are so important in the therapeutic process.

## Conclusion

The perspective of nurses on the remote therapeutic relationship with family members of patients diagnosed with COVID-19 infection perceived in the light of Peplau involved bonding and welcoming as points of trust development, which brought positive results to family members, given the uncertainties experienced during the pandemic.

Theassistive technologies use brings about the resolution of problems as well as improvements in the care process as it considers a broad view of the needs of the patient and their families. Furthermore, the development of assistance from a philosophical perspective expands the understanding and reflection necessary for the evolution of care.

Nursing is emerging with great relevance in the COVID-19 scenario and deserves to be the target of new studies in order to emphasize new care practices as well as the development of a holistic vision that includes the psychological and social needs of patients, with a great impact on their physical condition.
